# Late Onset Postpartum Eclampsia: It is Really Never Too Late—A Case of Eclampsia 8 Weeks after Delivery

**DOI:** 10.4061/2010/798616

**Published:** 2009-09-01

**Authors:** Jens Minnerup, Ilka Kleffner, Heike Wersching, Julian Zimmermann, Wolf-Rüdiger Schäbitz, Thomas Niederstadt, Rainer Dziewas

**Affiliations:** ^1^Department of Neurology, University of Münster, Albert-Schweitzer Straße 33, 48149 Münster, Germany; ^2^Department of Clinical Radiology, University of Münster, Albert-Schweitzer Straße 33, 48149 Münster, Germany

## Abstract

*Introduction*. Eclampsia is the combination of preeclampsia and seizures. Approximately one-half of all cases of eclampsia occur postpartum. Thereby late onset postpartum eclampsia is defined by its onset more than 48 hours after delivery. *Summary of Case*. We report a postpartum eclampsia occurring 8 weeks after delivery, which is the latest onset ever described. The course was complicated by an intracerebral hemorrhage (ICH). *Conclusion*. A late onset postpartum eclampsia even several weeks after delivery should be considered as possible diagnosis, since early treatment initiation with magnesium sulphate and antihypertensive medication prevents severe complications and reduces mortality.

## 1. Introduction

Preeclampsia is defined as the new onset of hypertension and proteinuria after 20 weeks of gestation in a previously normotensive woman [1]. The occurrence of generalized convulsions in a woman with preeclampsia without an alternative identifiable cause is referred to eclampsia. Approximately one-half of all cases of eclampsia occur postpartum [2, 3]. For a long time eclampsia was thought to not occur later than 48 hours after delivery [4]. This opinion was modified due to the onset of postpartum eclampsia occurring more than 48 hours after the onset of the postpartum period in some patients [5, 6]. Therefore late postpartum eclampsia (LPE) can be distinguished from early onset postpartum eclampsia by an onset later than 48 hours after term [5]. The latest onset of a LPE that has been reported in the literature so far was observed 23 days postpartum [5]. Characteristic findings on brain MRI include reversible white matter hyperintensities in T2-weighted images along with normal diffusion weighted imaging (DWI), thus representing a vasogenic edema [7, 8]. Usually, affected areas observed in the MRI are the parieto-occipital lobes, and less frequently other areas such as the frontal and the temporal lobe or the basal ganglia [9]. Here we report a postpartum eclampsia with the latest onset ever described, occurring 8 weeks after delivery. This case was complicated by an ICH due to the initially false diagnosis of an ischemic stroke and the consequently tolerated high blood pressure. 

## 2. Case Report

**Figure 1:**
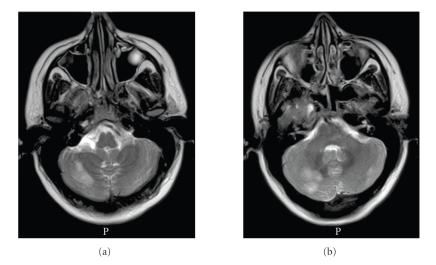
Axial T2-weighted MRI shows areas of hyperintense signal in both the right and left cerebellar hemispheres. Findings in this MRI had led to the initial false diagnosis of an ischemic stroke in the posterior circulation.

**Figure 2:**
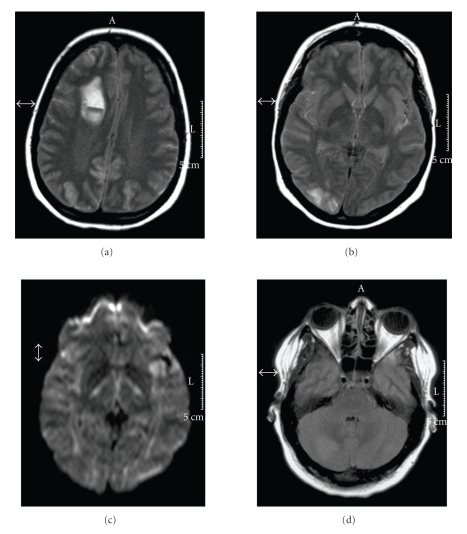
Axial FLAIR sequences show an ICH in the right frontal lobe (a). The combination of hyperintensities rostral of the ICH and in the right occipital lobe (b) with a normal DWI (c) in these areas is consistent with a vasogenic edema. The alterations in the cerebellum were largely unchanged (d).

**Figure 3:**
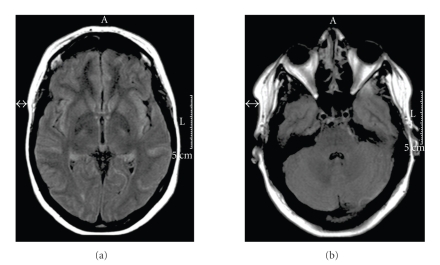
A followup MRI examination three months after admission shows a complete resolution of the vasogenic edema.

A 30-year-old woman (gravida five, para five) having a migraine with aura in her medical history delivered a healthy girl in pregnancy week 38 by caesarean section. During her antenatal period, she developed edema of the legs and gestational diabetes. She was normotensive and had no proteinuria throughout pregnancy and during the postpartum course. On postpartum day 53, she complained of a rapidly developing severe headache and a transient visual loss in her left visual field. She was admitted to a local hospital. Her neurological examination there was normal except for a left-sided hemianopsia. The blood pressure was 180/90 mmHg. A CT scan of the head was performed immediately after admission and revealed hypodensities in the cerebellum. An MRI scan of the brain showed areas of hyperintense signal on T2-weighted images in the right rather than in the left cerebellar hemisphere (Figure 1). A lumbar puncture and a transesophageal echocardiogram were normal. The diagnosis of an ischemic stroke in the posterior circulation was made. Three days after admission, the patient experienced two generalized tonic-clonic seizures. A CT scan showed an ICH in the right frontal lobe. The patient was transferred to our neurological intensive care unit for further treatment of the ICH. On admission here, neurological examination revealed a somnolent state, a bilaterally reduced vision, and a left-sided moderate hemiparesis. Brain MRI and magnetic resonance arteriography/venography (MRA/MRV) showed an ICH (Figure 2(a)) of the right frontal lobe, hyperintensities on FLAIR sequences (Figure 2(b)) of the right frontal lobe rostral of the ICH and in the right occipital lobe, whereas DWI (Figure 2(c)) revealed no abnormalities, thus suggesting a vasogenic edema in these areas. There was no evidence for an intracranial sinus thrombosis on MRV. Urinanalysis indicated a proteinuria. A diagnosis of LPE was made. Treatment with intravenous magnesium was started and supplemented with valproic acid (VPA), which was subsequently changed to levetiracetam. No further seizures occurred. As her blood pressure was still increased, she was treated with urapidil. During her hospital stay, pulsoxymetry detected decreased oxygen saturation without symptoms of respiratory distress. The CT pulmonary angiography revealed subsegmental pulmonary embolism. A laboratory screening for thrombophilia was performed and a positive lupus anticoagulant test could be found. The patient was discharged four weeks after admission without any sequelae. A subsequent repeat MRI (Figure 3) of the brain three months after initial admission showed, apart from residues of the ICH, a complete resolution of the previous abnormalities. 

## 3. Discussion

Important differential diagnoses of cerebellar lesions include multiple sclerosis and other inflammatory-demyelinating diseases, ischemic stroke, and neoplastic diseases [10]. Before referral to our hospital, the hyperintense lesions on the T2-weighted images in the posterior circulation were interpreted as stroke. However, no diffusion-weighted studies were done to corroborate the diagnosis. Furthermore, the arrangement of lesions was not consistent with an arterial supply. An eclampsia was initially not considered due to the long time interval between delivery and first symptoms even though the rapidly developing severe headache and the cortical visual deficit are typical symptoms [11]. In our department, the diagnosis of eclampsia was based on the clinical course, the detection of a vasogenic edema in absence of any cytotoxic edema on the MRI scan, and the combination of increased blood pressure with proteinuria and a recent childbirth in the patient's medical history. Frequent diseases causing seizures in puerperal period were ruled out. Particularly, there were no signs of a venous sinus thrombosis in the MRV. Other conditions causing seizures, such as meningitis and encephalitis, space-occupying lesions, and electrolyte or endocrine disturbances were also excluded. In addition, MRA, laboratory tests, and cerebrospinal fluid examination revealed no evidence for a vasculitis. Our patient had a pulmonary embolism and a positive lupus anticoagulant test, which is of interest, since thrombophilia and antiphospholipid antibodies have been found to be significantly associated with preeclampsia [12].

Complications in eclampsia are common and include acute renal failure, acute liver failure, and respiratory complications, such as aspiration pneumonia and acute pulmonary edema [13]. Mortality in eclampsia is mostly related to ICH [14]. Because of the severe complications, the appropriate therapy of eclampsia should be initiated as early as possible. For preventing and treating seizures in eclamptic patients magnesium sulphate is the drug of choice, as it is associated with a significant reduction of recurrent seizures, the risk for pneumonia, and admission to an intensive care unit [15, 16]. Mortality rates were also found to be substantially lowered compared to diazepam and tended to be reduced compared to phenytoin treatment [15, 16]. This is quite remarkable, since neurologists have a tendency toward using these classic anticonvulsants in patients with seizures that are due to other causes than eclampsia. Our patient was hypertensive with systolic blood pressures up to 200 mm Hg. According to the initial diagnosis of a posterior circulation infarction, the blood pressure was not lowered, as recommended by the national and international guidelines [17, 18] for the acute phase of an ischemic stroke. In eclampsia, the blood pressure value at which antihypertensive therapy should be initiated is not clearly determined due to the lack of clinical trials that address this issue. However, lowering blood pressure in eclamptic patients is recommended when systolic blood pressure reaches or exceeds 155–160 mm Hg, since higher values are associated with the occurrence of ICH [19].

This case emphasizes the importance of considering on late onset postpartum eclampsia even some weeks after delivery, as early diagnosis and subsequent initiation of the appropriate antihypertensive and anticonvulsant therapy prevent severe complications.
